# Usability and acceptability testing of a Plan of Safe Care in a mobile health platform

**DOI:** 10.3389/fpsyt.2023.1182630

**Published:** 2023-05-25

**Authors:** Krystyna R. Isaacs, Elina Bajracharya, Shantae Taylor, Katie Chang, Yukiko Washio, Trenee Parker, David A. Paul, Tony X. Ma

**Affiliations:** ^1^Benten Technologies, Manassas, VA, United States; ^2^ChristianaCare – Department of Pediatrics, Wilmington, DE, United States; ^3^Substance Use, Gender and Applied Research, RTI International, Research Triangle Park, Durham, NC, United States; ^4^Department of Obstetrics, Gynecology and Reproductive Sciences, Temple University Lewis Katz School of Medicine, Philadelphia, PA, United States; ^5^Delaware Division of Family Services, Department of Services for Children, Youth and Their Families, Wilmington, DE, United States; ^6^Sidney Kimmel Medical College at Thomas Jefferson University, Philadelphia, PA, United States

**Keywords:** substance use disorder, opioid use disorder, web-based case management, Agile, mHealth, contingency management, user-centered design, usability testing

## Abstract

**Purpose:**

Women who are pregnant or parenting while recovering from substance use disorder (SUD) are at risk for insufficient recovery support. With the federal mandate, implementation has been left to each state for the Plan of Safe Care (POSC), leading to challenges in providing comprehensive care coordination and meeting federal reporting requirements.

**Methods:**

This research tests the usability and acceptability of a POSC platform, called SAFE4BOTH, which combines a mobile health (mHealth) app for use by mothers with substance use disorder (MSUD) with a web-based case management system for use by stakeholders to reduce the issue of fragmented postnatal maternal and infant care. The platform was designed to enable access to services, improve reporting task workflow, and assist in improving interactions between mothers and service providers.

After applying a user-centered design approach, the usability and acceptability of the SAFE4BOTH platform were evaluated using focus groups, interviews, and a System Usability Scale (SUS). The evaluation involved four staff members from a Medication for Addiction Treatment clinic (comprising of three case management workers and one peer counselor), four state employees of the Delaware Division of Family Services, and 20 mothers with MSUD who had delivered infants in need of a POSC.

Features tested in the SAFE4BOTH platform included a secure, web-based POSC, a contingency management-based reward system, a micro-learning library, a resources locator, a chat messaging and videoconferencing system, a directory for contact management, a QR code reader, use of an appointment compliance system engaging geofencing, and an enhanced calendar. Family services and treatment center staff accessed SAFE4BOTH from their laptops or tablets, and MSUD accessed SAFE4BOTH from their phones.

**Results:**

Family services staff, treatment center staff, and MSUD participants rated SAFE4BOTH as usable and acceptable with average System Usability Scale scores of 68.1 (SD 8.5), 92.5 (SD 11.73), and 78.4 (SD 12.5) (respectively).

**Conclusion:**

The platform was judged both usable and acceptable by all three target populations (family services staff, treatment center staff, and MSUD). Further studies are planned to explore the efficacy of longitudinally supporting the mother’s recovery and the infant’s healthy development.

## Introduction

According to the 2021 National Survey on Drug Use and Health, approximately 16.4% of females aged 18–44 reported past-month illicit drug use, and 49.9% reported past-month alcohol use (1). Over 19,300 babies were diagnosed with neonatal abstinence syndrome/neonatal opioid withdrawal syndrome (NAS/NOWS) at birth during the same period (1–3). Aggregate hospital charges for NAS, a frequent result of opioid exposure, increased from $732 million to $1.5 billion in 2014, with 81% attributed to state Medicaid programs (4). Women who use illicit substances are likely also to be using alcohol and tobacco and struggling with traumatic personal histories (5). Additionally, they require comprehensive behavioral health care and coordination of services, especially during and after delivery (6–9). These mothers may also be more likely to experience emergency room visits and hospitalizations in the antenatal period and less likely to receive prenatal care (10).

The 2016 Comprehensive Addiction and Recovery Act and the recently updated Child Abuse Prevention and Treatment Act (11) require states to provide a Plan of Safe Care (POSC) for mothers who are at risk of relapse or unsafe conditions for their infants due to maternal substance use. With minimal requirements given by the federal government regarding the reporting and data requirements for the POSC, each state and local jurisdiction is responsible for creating its POSC. At a minimum, the states or jurisdictions must create and maintain a POSC to verify the mother is continuing her substance use disorder (SUD) care, confirm that infants are being discharged to a safe environment, and ensure the infants are being taken to regular well-baby visits (12). Each year, between 12,000 and 240,000 women are expected to need a POSC (13). However, the current state and local systems are not equipped to manage the unique challenges the federal mandate requires, and there is concern over how the law’s intended spirit of keeping infants and mothers safe will be enacted.

The level of oversight in a POSC requires significant funds, coordination, collaboration, and case management to facilitate integration across multiple state or local agencies, healthcare providers, and caretakers (8, 14). The economic burden of caring for mothers with SUD (MSUD) during and after pregnancy and their infants or children can be significant (15, 16). MSUD are at risk of insufficient support to provide care for their infants’ physical, emotional, and safety needs. Appointment compliance (17) can be a critical proxy measure to determine whether the mother is engaging in the healthcare system after delivery and progressing in her recovery.

Based on our qualitative work, a mobile health (mHealth) platform consisting of a web-based case management system for family services and treatment center staff and an app for the mothers (i.e., SAFE4BOTH) was developed to help MSUD adhere to their POSC plan after delivery. The goal of the SAFE4BOTH platform is to reduce fragmented prenatal and postnatal maternal and infant care for MSUD and improve interactions between MSUD and family services and treatment center staff. This study reports the findings of the usability testing of SAFE4BOTH with the target population of MSUD and staff at local and government agencies recruited in the mid-Atlantic region.

## Materials and methods

### Design

As described earlier, extensive formative research supported a user-centered design process completed with MSUD and family services and treatment center staff prior to the creation of the mHealth platform (18). It was determined that significant barriers to an efficient workflow were a lack of communication between family services staff, treatment staff, and the mothers, poor appointment adherence (frequently caused by a lack of transportation or childcare), and difficulties with maintaining updated contact information. Contingency management or rewards (points earned in exchange for items from a donation center in return for verified appointment adherence or completing educational materials) were very popular features with the mothers and, as such, were incorporated into the mHealth app design.

### Recruitment

Staff from the Delaware Division of Family Services-Department of Services for Children, Youth, and Their Families, and the Delaware-based CORAS Wellness & Behavioral Health (formerly Connections Treatment Center) organization were asked to use the web-based case management website component of the SAFE4BOTH platform for family services and treatment center staff. Mothers were approached within 24 h of delivery at the ChristianaCare (Newark, DE) Labor and Delivery, Pediatric, and Maternity Units if their infants were under observation for NAS/NOWS and were asked to use the mHealth app component of the SAFE4BOTH platform. MSUD were eligible to participate in the study if they met the following criteria: their age was between 18 and 44, they had delivered an infant who was diagnosed with NAS/NOWS and therefore required a POSC, were English speaking, residing in Delaware, were not ill and the infant was going to go home with the mother. ChristianaCare provided the IRB for this study.

### Usability testing

Staff members tested the platform’s ability to provide instant access to the current web-based POSC, to verify appointment attendance, easy access to federally-mandated automated summary reports for family services and treatment center facilities, in-app text messaging and videoconferencing, a video library with educational materials tailored to MSUD, an in-app contact list for all relevant stakeholders, and a contingency management mechanism to reward MSUD for viewing educational materials delivered on their mobile devices.

Usability testing for family services and treatment center staff was conducted with the SAFE4BOTH prototype in July–September 2021 using think-aloud (19) testing sessions at their facility. Each family services staff person (*n* = 4) or treatment center counselor (*n* = 4) was paired with program staff from Benten. During the testing session, Benten staff played the role of an MSUD who had recently given birth. A script was provided to assure that the most significant components of the case management within the SAFE4BOTH platform were tested over a single 60–90 min session and testers were asked to ‘think aloud’ as they were using the web-based case management system and comment on their thoughts of the design and processes as they used the Case Management System to complete the tasks requested in the script.

After completing eligibility questions and informed consent, MSUDs were asked to download and meet with the research coordinator to go through a script with a prescribed set of tasks. It could take up to three sessions to complete the intake and usability testing, depending on the time the mother had available while in the maternity ward. She was allowed to use the app and continue going through the script on her own between research coordinator visits. The MSUD script required the mothers to use in-app text messaging and videoconferencing, a video library with educational materials tailored to MSUD, an in-app contact list for all relevant stakeholders, and a contingency management system that included a wish list for future items to be purchased with points earned from watching the videos, taking quizzes and updating their contact information.

### Assessment instruments and usage measures

After the sessions, participants rated the design using the System Usability Scale (SUS) survey instrument (20, 21), which included three additional open-ended questions about the usability and acceptability of the design. The SUS is a widely used and validated scale; the scale can be employed to evaluate a variety of technologies (e.g., web and mobile applications) and provides a score to evaluate the perceived usability and acceptability of the two main components of SAFE4BOTH, namely the mobile app for mothers and web-based case management for staff.

Participation in the testing was voluntary and treatment center staff members were given $25 gift cards in compensation. After completion of the usability testing, MSUD completed a SUS survey which included three open-ended questions and were given a $60 gift card in compensation. Usage data was automatically captured from the app and obtained from the server to assess user engagement with SAFE4BOTH’s mobile app for mothers.

## Results

### Pilot testing with family services and treatment center staff of the web-based platform

**Figure 1:**
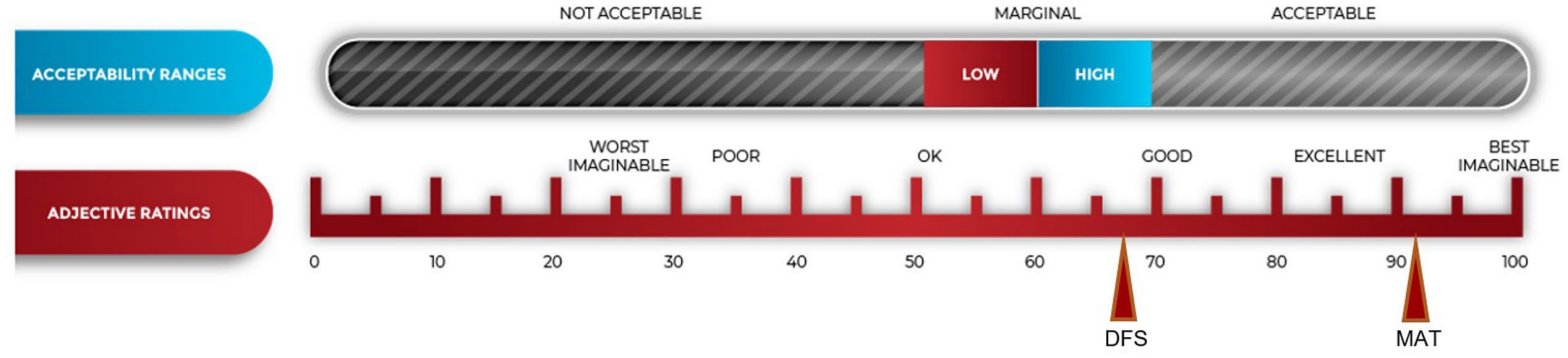
System usability scores (SUS) from family services and treatment center staff after usability testing.

A total of four family services and four treatment center staff completed the testing of the web-based case management system (see Table 1), and all participants completed a SUS to rate the prototype SAFE4BOTH platform. The family services staff rated the SAFE4BOTH’s case management platform system as “HIGH MARGINAL” for acceptance and usability (68.10, SD 8.50) and in the “OK” range for adjective ratings, while the treatment center staff rated the SAFE4BOTH platform as “ACCEPTABLE”—“BEST IMAGINABLE” range (92.50, SD 11.73) (see Figure 1).

**Table 1:** Demographics for family services and treatment center staff.

Group	Hispanic/Latino	Racial category
DFS staff (*n* = 4)	4 Non-Hispanic/Latino	3 White and 1 more than one race
MAT (*n* = 4)	4 Non-Hispanic/Latino	3 White and 1 Black

Although the family services staff members’ SUS scores were in the HIGH MARGINAL range, all four family services participants responded that they would want to use the platform if it was available, and this positive attitude is reflected in the representative comments displayed in Table 2.

**Table 2:** Representative family services and treatment center staff responses to open-ended questions after usability testing.

Delaware Division of Family Services (DFS) Staff	*I think the application is a great way to keep all the information organized and in one place and being able to see progress in the MAT programs. (DFS03)* [I liked the] *easy communication with moms and [being] able to track follow-up appointments (DFS08)* [I liked the] *time line on the dashboard (DFS04)* [I found it] *easy to use overall (DFS02)*
Medication for Addiction Treatment (MAT) Center Staff	[The SAFE4BOTH platform provides a] *central way to track who’s POSC has and has not been completed and* [is a] *MUCH easier way to fill out the POSC* versus *a word document which can be frustrating (MAT07)* *I love this system. I’m sure it will help us all communicate the needs of the mothers and babies much better as well! (MAT10)* [My three favorite features were the list of outstanding] *tasks, timeline and wish list (MAT04)* [I liked the] *ability to see if clients attended appointments (MAT05)*

### Pilot testing of the mobile health app with MSUD

Of the 94 women who were approached over the 17 months (March 2021–July 2022), 29 women completed the informed consent and were enrolled in the pilot. The reasons why some women were not enrolled included being COVID-positive at delivery (*n* = 3), no diagnosis of NAS in the infant (so no POSC was required, *n* = 5), out-of-state residence (*n* = 13), no fluency with English (*n* = 3), incarceration (*n* = 2) or no interest (*n* = 5). Numerous women expressed an interest at the first contact but then failed to respond to future outreach attempts (*n* = 27). Eight of nine women who did not complete the testing did not return phone calls or in-app text messages. Three attempts over 2 weeks were made to re-engage the mothers, at which point they were dropped from the study. One mother reached back out to the research coordinator and informed her that she was no longer interested in participating and so was marked as ‘withdrawal’. As such, of those 29 who were enrolled, 20 completed the pilot study. The demographics of the women who completed the pilot testing are in Table 3.

**Table 3:** Demographic information for MSUD participants.

	Percent
Race/ethnicity	
White	14/20 (70%)
Black or African American	6/20 (30%)
Non-Hispanic/Latino	19/20 (95%)
What is the highest grade you finished in school or through home-schooling?	
Grades 9–11	4/20 (20%)
High school graduate (12th grade)	7/20 (35%)
Junior college degree	1/20 (5%)
Some college	7/20 (35%)
Some post-college work	1/20 (5%)
Total	20 (100%)
Age group	
25–29	3/20 (15%)
30–34	13/20 (65%)
35–39	3/20 (15%)
40–44	1/20 (5%)
Total	20 (100%)
Number of mothers with more than 1 child	18/20 (90%)

**Figure 2:**
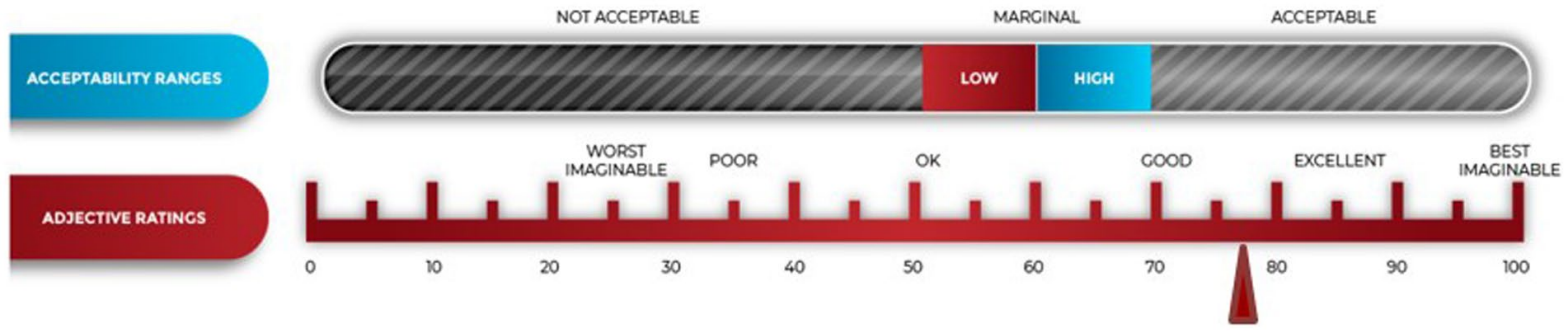
System usability scores from mothers with SUD.

MSUD participants were given a script for activities to complete. Out of the ten features of the application listed in the script, all participants (*n* = 20) completed the testing script, used the videoconferencing feature with the assigned staff, and then completed a SUS. The MSUD participants gave the application a SUS of 78.4 (SD 12.5), which is equivalent to an ACCEPTABLE-GOOD rating (see Figure 2).

A closer look at the specific SUS responses revealed that an overwhelming majority of the women found the application easy to use (95%) and were confident they could master its features (90%). In addition, 75% were looking forward to using this application in the future and 80% would consider downloading the application if it were available (see Table 4).

**Table 4:** A detailed review of the MSUD participants’ SUS responses after usability testing.

Question	Strongly agree/agree	Neutral	Strongly disagree/disagree
I think that I would like to use this mobile application frequently	15 (75%)	2 (10%)	3 (15%)
I thought this mobile application was easy to use	19 (95%)	1 (5%)	0
I would imagine that most people would learn to use this mobile application	19 (95%)	0	1 (5%)
I felt very confident using this mobile application	18 (90%)	2 (10%)	0
I would consider downloading the mobile application if it were available	16 (80%)	2 (10%)	2 (10%)

A review of the open-ended questions where the women were asked to list the three features they liked most about the app supported the conclusion that the women saw this application as valuable and useful (see Table 5).

**Table 5:** MSUD response to “Top 3 most liked app features.”

Most liked app features	Mentioned in the free response section
Education and resource materials	12 (60%)
Contacts/appointments /calendars	6 (30%)
Reward system	5 (25%)
Enhanced communication	4 (20%)
Easy access to the POSC	2 (10%)

The most popular feature was the educational materials, followed by the ability to easily schedule appointments using the contacts and calendar elements and the built-in reward system. Several mothers identified the enhanced communication options through the chat and video call features as important to them. Two specifically mentioned that easy access to their POSC was one of the top three critical features they liked in the app. Additional responses related to what the women would like to see improved in the app (such as more options in the rewards “shop”, additional features in the text and video chat elements, and more content in the microlearning/resources section) will be used to drive the revisions to the design of the app in future iterations.

The MSUD responses to the open-ended questions in the SUS reflected their positive attitude towards the app in general (see Table 6).

**Table 6:** Representative MSUD responses to open-ended questions in the SUS after usability testing.

Mothers with substance use disorder	*I like how you can chat with providers (Mom28)* *Organized (Mom27)* *Important contacts and appointments in one place (Mom11)* *Offers a lot of help/organization/answers to questions (Mom13)* *The helpful videos (Mom24)* *I think the rewards points program will influence users to do more (Mom11)* *Ability to get points and spend them (Mom13)* *That you could look up the information in regards to your safety plan (Mom25)* *I’m learning more about the process of my child safety and health (Mom14)*

## Discussion

### Overview

The current report describes the newly developed SAFE4BOTH platform and usability and acceptability testing with family service and treatment center staff members and MSUD. The SAFE4BOTH platform was rated usable and acceptable by both MSUD as well as the family services and treatment center staff members. The platform received OK to EXCELLENT ratings for its ability to incorporate educational materials as well as digital and feature-rich case management mechanisms combined with a mobile-based POSC with a reward system to improve coordination of care for women who are in recovery with infants exposed to substance use during gestation. The MSUD scored the app as ACCEPTABLE for usability and GOOD in the adjective ratings. Their open-ended responses cited the value of the calendar, communications, and organization. Only two of the 20 MSUDs said they would not download the app if it were available today. Only one expressed concern about difficulties navigating the mHealth app while using it. Several women suggested improvements that will be incorporated into the next version related to making the chat function more user-friendly.

The treatment center staff rated the case management component of the SAFE4BOTH platform as ACCEPTABLE and gave it the BEST IMAGINABLE adjective rating. They repeatedly asked when the platform could be rolled out for use and were excited about the enhanced communication and the ability to monitor appointment compliance. The family services staff rated the acceptability as MARGINAL-HIGH and gave the platform an OK rating. They saw the platform’s potential as noted in their comments but seemed reluctant to adopt another notation or tracking system to use with the mothers with SUD. It is unclear at this time why the treatment center staff members scored the platform so much higher than the family services staff. Future research will focus on determining what would make the platform more attractive to family services workers. One possibility for increasing satisfaction with the platform is that when the system is fully integrated with the family service staff’s existing case-management software, the need for double data entry will be removed. In addition, when the SAFE4BOTH web-based POSC is readily accessible to all healthcare providers, state-based child welfare employees, and mothers, it will be possible to fill out a large portion of the POSC before delivery when the mother is at a treatment center clinic and can work with a pregnancy counselor, rather than shortly after delivery with a family services staff person. Combining these two additional features (integration and expanded access) will allow for the existence of a substantially pre-filled POSC available for updating at the time of hospital discharge and is expected to significantly reduce the family services staff members’ workload. SAFE4BOTH has the potential to greatly improve care transitions from medication for addiction treatment centers for mothers with SUD to birth hospitals to homes (22).

### Revisions to the original pilot protocol

Due to the COVID pandemic and logistical issues related to testing in a clinical setting, modifications had to be made to the original pilot protocol. Case managers at family services and the counselors at the treatment clinics found it very difficult to add platform development testing to their already demanding schedules. In addition, the State of Delaware information technology department’s requirements for personal information protection put significant limitations on the usability testing phase. As such, it was determined that for the usability testing, the best approach was to do separate sessions where developers interacted with the users to test the system features using a think-aloud script.

Accessing and recruiting mothers in the hospital on the post-partum floor during the COVID-19 pandemic was also extremely difficult. Frequently, during the height of the pandemic, the mother was discharged within 24 h of delivery while her infant stayed in the hospital under observation. The research study design was revised such that it was possible to brief the mothers who were in treatment at the clinic associated with the hospital before delivery. Then enrollment, follow-up, and testing were completed when the mothers were visiting their infants.

A final unexpected technical challenge was the inability to download the SAFE4BOTH app to the mother’s phone while she was in the hospital. Although the hospital provides free guest Wi-Fi, the bandwidth was too limited to download the app to the mothers’ phones. A Wi-Fi system was purchased just for this study to bypass the hospital settings and guest Wi-Fi limitations.

Despite recruitment and follow-up complications due to the COVID-19 pandemic, there were positive outcomes derived from conducting the study during the pandemic. When videoconferencing was initially proposed to meet with the mothers, the family services staff were highly reluctant to adopt this technology rather than in-person home visits. After the pandemic began, they were much more open to the idea. Similarly, while delivering educational content via a mobile phone was considered favorable in the initial stages, it was much more enthusiastically embraced after all in-person educational classes were canceled at the treatment center clinic.

### Limitations

There were several limitations to the study design. For this stage of usability testing, the study could only include MSUD who lived in the State of Delaware, as the POSC is specific to the state in which it is implemented. This exclusion criterion excluded mothers from surrounding states such as Pennsylvania. In addition, the study utilized a short usability testing period, typically restricted to the use of a script with a study coordinator in a 1–2 h testing period. In normal circumstances, the app would be expected to be used over a 12-month pre-and post-natal period. As with most app usability studies, the number of MSUD testers was limited to 20. While 20 participants can usually identify 90%–95% of all flaws in an app (23), further testing with larger group sizes will be planned before the product’s release.

## Conclusion

SAFE4BOTH is among the first mHealth apps with a comprehensive platform that provides integrated care coordination to be used by MSUD, any provider, and staff from state and local government agencies. The utilization of a secure, web-based POSC with a mobile app such as the SAFE4BOTH platform is feasible by families, providers, and child protection agencies and can provide incentives for mothers with SUD to continuously engage in care. By enhancing communication within and between organizations attempting to provide care and support for mothers with SUD, care can be focused on supporting recovery and providing a safe environment. Large population-based studies will be needed to determine if SAFE4BOTH can reduce the risks of adverse outcomes in this high-risk population.

## Data availability statement

The raw data supporting the conclusions of this article will be made available by the authors, without undue reservation.

## Ethics statement

The studies involving human participants were reviewed by ChristianaCare IRB. The patients/participants provided their written informed consent to participate in this study.

## Author contributions

KI, EB, ST, YW, DP, KC, and TM contributed to the design of the experiment, participated in the research, and edited the manuscript. TP advised on the design of the original protocol and adaptations to the protocol during the testing. All authors contributed to the article and approved the submitted version.

## Funding

This research was funded through the National Institutes of Health-National Institute on Drug Abuse Grant (# R43DA048673) and the Health Resources and Services Administration Maternal and Child Health Bureau’s Challenge Grant Award titled “Addressing Opioid Use Disorder in Pregnant Women and New Moms.”

## Conflict of interest

TM and KC are co-owners of Benten Technologies, the company that is designing this system and will eventually market the SAFE4BOTH product.

The remaining authors declare that the research was conducted in the absence of any commercial or financial relationships that could be construed as a potential conflict of interest.

## Publisher’s note

All claims expressed in this article are solely those of the authors and do not necessarily represent those of their affiliated organizations, or those of the publisher, the editors and the reviewers. Any product that may be evaluated in this article, or claim that may be made by its manufacturer, is not guaranteed or endorsed by the publisher.
